# Efficacy of ESHAP versus ICE plus dexamethasone (DICE) as salvage chemotherapy for relapsed or refractory diffuse large B-cell lymphoma

**DOI:** 10.1080/07853890.2023.2261109

**Published:** 2023-09-25

**Authors:** Pattamaporn Boonlerd, Adisak Tantiworawit, Lalita Norasetthada, Chatree Chai-Adisaksopha, Teerachat Punnachet, Nonthakorn Hantrakun, Pokpong Piriyakhuntorn, Thanawat Rattanathammethee, Sasinee Hantrakool, Ekarat Rattarittamrong

**Affiliations:** Department of Internal Medicine, Faculty of Medicine, Chiang Mai University, Chiang Mai, Thailand

**Keywords:** Relapsed or refractory diffuse large B-cell lymphoma, diffuse large B-cell lymphoma, lymphoma, ICE chemotherapy, ESHAP chemotherapy, DICE chemotherapy

## Abstract

**Objectives:**

To compare the efficacy and side effects of salvage chemotherapy between etoposide, methylprednisolone, cytarabine and cisplatin (ESHAP) and ifosfamide, carboplatin and etoposide plus dexamethasone (DICE) for relapsed or refractory diffuse large B-cell lymphoma (DLBCL).

**Methods:**

Medical records of patients with relapsed or refractory DLBCL receiving second-line ESHAP or DICE chemotherapy with or without rituximab from January 2007 to November 2022 were retrospectively reviewed. The primary objective was progression-free survival (PFS). The secondary objectives were overall survival (OS), overall response rate (ORR) and adverse events (AEs).

**Results:**

Seventy patients were enrolled including 21 patients who received ESHAP and 49 patients who received the DICE regimen. Six patients (28.6%) and 19 patients (38.8%) in the ESHAP and DICE groups underwent ASCT, respectively. The ORR was 47.6% for ESHAP and 53.1% for DICE (*p* = .67). The two-year PFS was 14.3% for ESHAP and 26.5% for DICE (*p* = .33) with median PFS of 5 months and 14 months, respectively (hazard ratio 0.74; 95% CI 0.39–1.36, *p* = .330). The two-year OS was 14.3% for ESHAP and 26.5% for DICE (*p* = .37) with median OS of 8 months and 19 months, respectively. Patients in ESHAP group have more all-grade renal impairment than DICE group (23.8% vs. 6.1%, *p* = .047).

**Discussion and conclusions:**

Efficacy between ESHAP and DICE regimens as salvage chemotherapy for relapsed or refractory DLBCL was not significantly different in terms of two-year PFS, two-year OS and ORR. DICE regimen had less renal AE than ESHAP.

## Introduction

Diffuse large B-cell lymphoma (DLBCL) is the most common type of aggressive lymphoma and accounts for about 30% of non-Hodgkin lymphoma (NHL) [1]. Rituximab in combination with cyclophosphamide, doxorubicin, vincristine and prednisolone (R-CHOP) is the standard treatment and has the potential to cure more than 60% of DLBCL patients [2]. However, approximately 10–15% of patients with DLBCL have refractory disease, while 20–25% suffer from relapsed disease [1]. In patients with chemotherapy-sensitive relapsed or refractory disease, salvage chemotherapy with autologous stem cell transplantation (ASCT) offers the best chance of cure [1,3]. The frequently used salvage regimens are ICE (ifosfamide, carboplatin and etoposide) [4,5], DHAP (dexamethasone, high dose cytarabine and cisplatin) [6], ESHAP (etoposide, methylprednisolone, high dose cytarabine and cisplatin) [7] and GDP (gemcitabine, dexamethasone and cisplatin) [8] with or without rituximab. According to the Collaborative Trial in Relapsed Aggressive Lymphoma (CORAL) study, which aimed to evaluate the choice of salvage chemotherapy in relapsed and refractory DLBCL between DHAP and ICE in combination with rituximab (R-DHAP or R-ICE), the efficacy of both regimens was comparable in terms of response rate and survival outcomes. R-DHAP had more grades 3–4 non-hematologic adverse events (AEs) especially renal toxicity than R-ICE [9]. In addition, ICE can be administered as either outpatient-based fractionated ICE or inpatient-based conventional ICE [10].

Limited data compare the effectiveness of salvage chemotherapy between ESHAP and ICE in patients with relapsed or refractory DLBCL. Hence, the purpose of this research was to evaluate ESHAP and ICE plus dexamethasone (DICE) regimens in patients with relapsed or refractory DLBCL regarding the response rate, progression-free survival (PFS), overall survival (OS) and AEs.

## Materials and methods

This study was a retrospective cohort study approved by the Institutional Review Board (IRB) of the Faculty of Medicine, Chiang Mai University (study code MED-2564-08234 and certificate number 265/2021). The requirement for consent was waived by the ethics committee. Eligible patients were aged 18 years or older diagnosed with relapsed or refractory DLBCL and received salvage chemotherapy including ESHAP or DICE (both conventional and fractionated DICE) as a second-line treatment at Maharaj Nakorn Chiang Mai Hospital from January 2007 to November 2022. Either patients who subsequently underwent ASCT or only received salvage chemotherapy without ASCT were eligible. Patients who received treatment with ESHAP or DICE as another line of salvage chemotherapy regimen had a history of HIV infection, had concurrent cancer, were pregnant or were breastfeeding were excluded. The ESHAP regimen consisted of etoposide 40 mg/m^2^ by intravenous (IV) on days 1–4, methylprednisolone 500 mg by IV on days 1–5, cytarabine 2 g/m^2^ by IV over two to three hours on day 5, and cisplatin 25 mg/m^2^ by IV 24-h infusion on days 1–4. Conventional DICE chemotherapy was administered as follows: etoposide 100 mg/m^2^ by IV bolus on days 1–3; carboplatin area under the curve (AUC) 5 (5 × [25 + CrCl], maximum dose 800 mg) by IV bolus on day 2; and ifosfamide admixed with mesna both at a dose of 5 g/m^2^ by 24-h continuous infusion beginning on day 2; dexamethasone 40 mg/day on days 1–5 by oral or IV. Ifosfamide was divided to 1670 mg/m^2^ by IV on days 1–3 in fractionated DICE regimen. Rituximab 375 mg/m^2^ by IV on day 1 was added to ESHAP or DICE for accessible patients who did not receive rituximab as induction therapy or prior received R-CHOP but had late relapsed disease. The chemotherapy was given every 21–28 days.

Patients who achieved a complete response (CR) or partial response (PR) after the second to the fourth cycle of salvage therapy were given high dose conditioning regimen with carmustine 300 mg/m^2^ on day −6, etoposide 200 mg/m^2^ on days −5 to −2, cytarabine 200 mg/m^2^ on days −5 to −2, and melphalan 140 mg/m^2^ on day −1 (BEAM) and ASCT.

Filgrastim 5 mcg/kg/day once daily was prescribed in all patients for 7–11 days depending on the febrile neutropenia risk of individual patients. Filgrastim was increased to 10 mcg/kg/day following the third or fourth cycle until the completion of peripheral blood stem cell collection. Antimicrobial prophylaxis with acyclovir 400 mg twice daily (for herpes zoster infection), co-trimoxazole (400/80) two tablets twice weekly (for *Pneumocystis jirovecii* infection), fluconazole 200 mg once daily (for fungal infection) were prescribed in all patients except who had contraindications. The patients who had chronic hepatitis B viral (HBV) infection (HBsAg or anti-HBc positive) received lamivudine or tenofovir for prevention of HBV reactivation as an institutional guideline.

The response was assessed using the 2014 Lugano criteria [11] since PET-CT was not accessible for all patients. The relapsed disease was further subdivided by relapsing onset as relapse within 12 months (early relapse) and relapse after 12 months (late relapse).

### Objectives and endpoints

The primary objective was to evaluate a two-year PFS in each regimen, which was defined as the interval between the start of salvage chemotherapy and the onset of the first disease progression or death from any cause. The secondary objective was to assess the two-year OS, which was defined as the time between first receiving salvage chemotherapy and death from any cause and response rate in each regimen. Another secondary objective was AEs, which were divided into hematologic AE and non-hematologic AE. The criteria and grading of AEs were based on common terminology criteria for adverse events (CTCAE) version 5.0 [12].

### Statistical analysis

The estimated sample size used a test of non-inferiority. The previous study [13] showed that the probability of death in relapsed or refractory DLBCL patients who received ESHAP and ICE was 0.55 and 0.40, respectively. The sample was then divided into the ratio of 2:3 between ESHAP and DICE, respectively. Assuming that the events were independent and identically distributed, the expected number of events in each group was calculated. A total of 70 patients were needed for the trial, with 28 patients in the ESHAP group and 42 patients in the DICE group.

Statistical analyses were performed using Stata 17 (StataCorp, College Station, TX). Statistics and analysis were calculated in both groups in terms of age, gender, a subtype of lymphoma according to Hans’ algorithm [14], Ann-Arbor stage, International Prognostic Index (IPI), pretreatment lactate dehydrogenase (LDH) level, previous chemotherapy, rituximab combination and treatment response (refractory, early relapse, late relapse disease). The DICE regimen group was also divided into conventional and fractionated DICE. The quantitative data were presented in mean ± SD, median (range) or percentage. Comparisons of clinical characteristics and AEs between groups were performed using Chi-square and Fisher’s exact test for categorical data and Student’s *t*-test or Mann–Whitney’s *U*-test for continuous data. Univariable Cox proportional hazard regression models were used to perform an association of factors related to PFS and OS. The differences in response to each treatment were statistically compared. The level of statistical significance was set at *α* = 0.05. Survival outcomes were demonstrated using the Kaplan–Meier method, and groups were tested using log-rank analysis with a statistical significance level of *α* = 0.05.

## Results

### Clinical characteristics

Overall, 70 patients with relapsed or refractory DLBCL were included. Twenty-one patients received the ESHAP regimen (30%) and 49 patients received the DICE regimen (70% with 75.5% receiving fractionated DICE). The median age was 54.8 years and 44 patients (62.7%) were men. Sixty-four cases (91.4%) were classified as non-germinal centre B-cell (non-GCB) subtypes. Forty-four cases (62.9%) received CHOP as a first-line therapy including 12 patients (57.1%) and 32 patients (65.3%) in ESHAP and DICE group while six cases (28.6%) and 15 cases (30.6%) in ESHAP and DICE group previously received R-CHOP, respectively. Sixteen patients (22.8%) received a rituximab combination with a salvage regimen including 4 patients (19.0%) and 12 patients (24.5%) in ESHAP and DICE groups. According to disease status, 20 patients (28.6%) had refractory disease, 25 patients had early relapsed disease (35.7%), and 25 patients had late relapsed disease (35.7%). Six patients (28.6%) and 19 patients (38.8%) in ESHAP and DICE groups underwent ASCT, respectively. The clinical characteristics of patients in both groups are shown in Table 1.

**Table 1.** Clinical characteristics of the patients.

Clinical characteristics	Total *N* = 70	DICE *N* = 49 (70%)	ESHAP *N* = 21 (30%)	*p* Value
Median age (±SD)	54.88 ± 12.88	56.14 ± 12.31	51.94 ± 13.97	
Age ≥60 years	32 (45.7%)	25 (51.0%)	7 (33.3%)	.173
Male	44 (62.7%)	29 (59.2%)	15 (71.4%)	.331
Subtype				
GCB	5 (7.1%)	4 (8.2%)	1 (4.8%)	1.000
Non-GCB	64 (91.4%)	44 (89.8%)	20 (95.2%)	
Ann Arbor stage I–II	20 (28.6%)	14 (28.6%)	6 (28.6%)	1.000
Ann Arbor stage III–IV	50 (71.4%)	35 (71.4%)	15 (71.4%)	
Extra-nodal involvement				
0–1	46 (66.7%)	31 (64.6%)	15 (71.4%)	.579
≥2	23 (33.3%)	17 (35.4%)	6 (28.6%)	
LDH rising	58 (82.9%)	43 (87.8%)	15 (71.4%)	.097
Bulky	27 (38.6%)	19 (38.8%)	8 (38.1%)	.957
IPI				.202
Low	9 (12.9%)	4 (8.1%)	5 (23.9%)	
Low intermediate	21 (30.0%)	17 (34.7%)	4 (19.0%)	
High intermediate	18 (25.7%)	14 (28.6%)	4 (19.0%)	
High	22 (31.4%)	14 (28.6%)	8 (38.1%)	
Prior treatment				
CHOP	44 (62.9%)	32 (65.3%)	12 (57.1%)	.351
R-CHOP	21 (30.0%)	15 (30.6%)	6 (28.6%)	
Other^a^	5 (7.1%)	2 (4.0%)	3 (14.3%)	
Rituximab combination	16 (24.6%)	12 (37.3%)	4 (19.0%)	.551
Relapsed/refractory				
Refractory disease	20 (28.6%)	15 (30.6%)	5 (23.8%)	.846
Early relapse disease	25 (35.7%)	17 (34.7%)	8 (38.1%)	
Late relapse disease	25 (35.7%)	17 (34.7%)	8 (38.1%)	
Patients undergoing ASCT	25 (35.7%)	19 (38.8%)	6 (28.6%)	.414
Year of receiving chemotherapy				
2007–2015	5 (7.1%)	4 (8.1%)	1 (4.8%)	.525
2016–2022	65 (92.7%)	45 (91.8%)	20 (95.2%)	

ESHAP: etoposide, methylprednisolone, high-dose cytarabine, cisplatin; DICE: dexamethasone, ifosfamide, carboplatin, etoposide; GCB: geminal centre B-cell; non-GCB: non-geminal centre B-cell; LDH: lactate dehydrogenase; IPI: International Prognostic Index; CHOP: cyclophosphamide, doxorubicin, vincristine, prednisolone; R-CHOP: rituximab, cyclophosphamide, doxorubicin, vincristine, prednisolone; ASCT: autologous stem cell transplant.

^a^Other regimens: VP: vincristine, prednisolone; CVP: cyclophosphamide, vincristine, prednisolone; CEOP: cyclophosphamide, etoposide, vincristine, prednisolone.

### Survival rate and response to treatment

**Figure 1.**
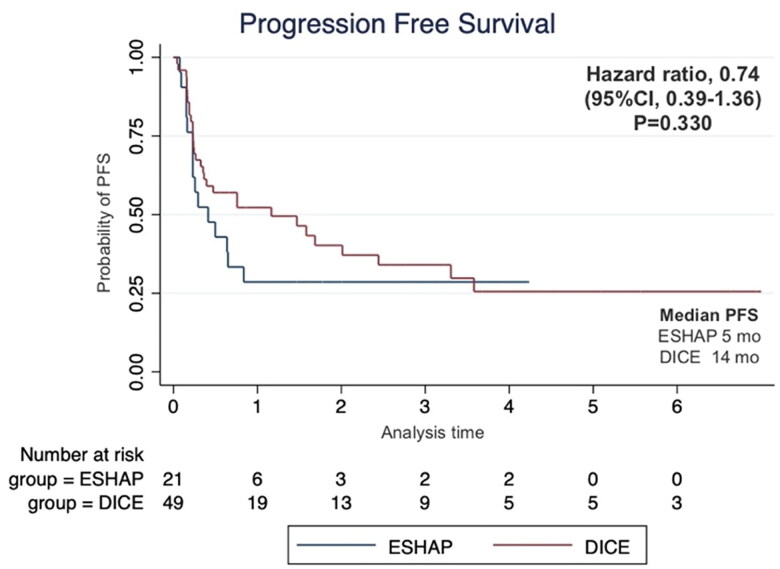
Kaplan–Meier’s curve of progression-free survival according to salvage chemotherapy.

**Figure 2.**
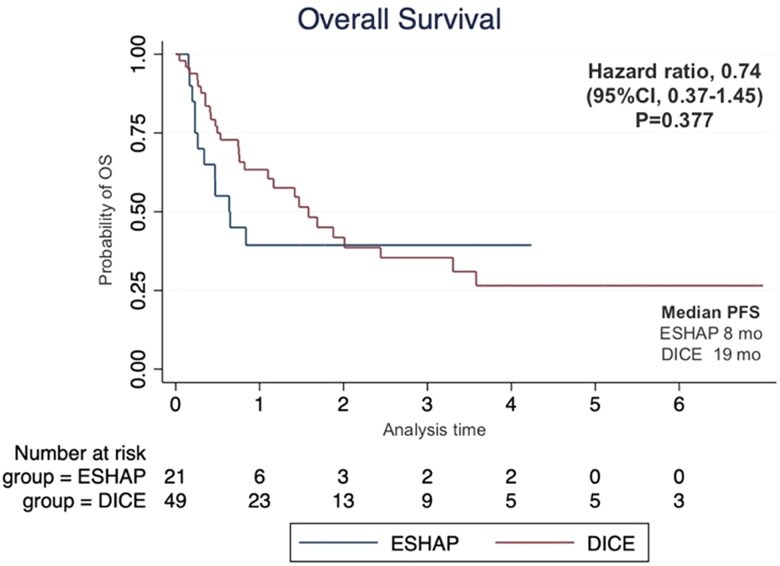
Kaplan–Meier’s curve of overall survival according to salvage chemotherapy.

**Figure 3.**
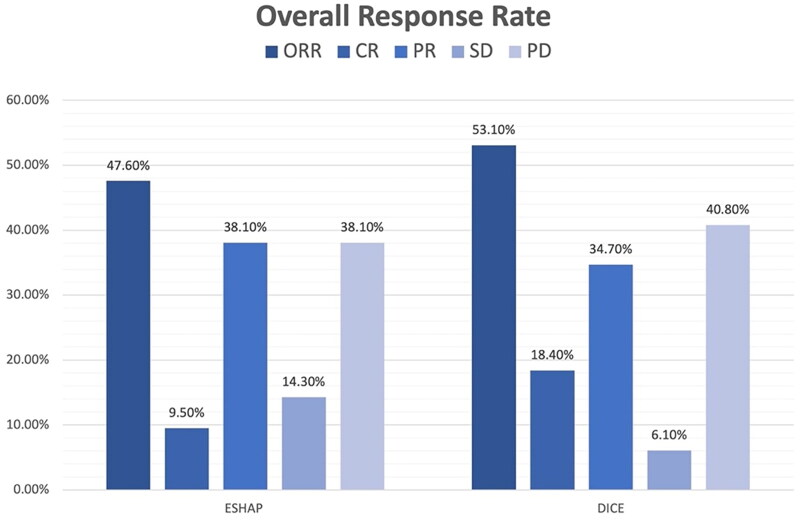
Response rate of salvage chemotherapy.

The median follow-up time was 17 months. The two-year PFS was 14.3% for ESHAP and 26.5% for DICE (*p* = .33) with median PFS of 5 months and 14 months, respectively (Figure 1). The two-year OS was 14.3% for ESHAP and 26.5% for DICE (*p* = .37) with median OS of 8 months and 19 months, respectively (Figure 2). The overall response rate (ORR) was 47.6% for ESHAP and 53.1% for DICE (*p* = .67) with CR rates of 9.5% and 18.4%, respectively (Figure 3). The combination of rituximab and the DICE regimen resulted in a greater probability of achieving response (CR or PR) (91.6%), while the use of DICE regimen alone had an ORR of 40.5%.

### Conventional DICE vs. fractionated DICE

The median PFS of conventional DICE and fractionated DICE was 20 months and 5 months, respectively (*p* = .37). While the median OS of conventional DICE and fractionated DICE was 20 months and 17 months, respectively (*p* = .72). The ORR of conventional DICE and fractionated DICE was 91.6% and 40.5% (*p* = .002) with CR rates of 16.7% and 18.9%, respectively (*p* = .005).

Rituximab was combined in 50% of the patients (six out of 12) who received conventional DICE and in 16.2% (six out of 37) who received fractionated DICE (*p* = .04). The ORR of conventional DICE and fractionated DICE with rituximab combination was 100.0% and 83.3% (*p* = 1.000) with CR rates of 16.7% and 33.3%, respectively (*p* = .545) as shown in Table 2.

**Table 2.** Response rate of salvage chemotherapy according to the rituximab combination.

	ORR	*p* Value	CR	*p* Value
ESHAP				
ESHAP (*N* = 17)	9 (53.0%)	.586	2 (11.8%)	.644
R-ESHAP (*N* = 4)	1 (25%)		0 (0%)	
DICE				
Conventional DICE (*N* = 12)	11 (91.7%)	.002	2 (16.7%)	.005
Fractionated DICE (*N* = 37)	15 (40.5%)		7 (18.9%)	
Rituximab combination in				
Conventional DICE (*N* = 6)	6 (100%)	.047	1 (16.7%)	.545
Fractionated DICE (*N* = 6)	5 (83.3%)		2 (33.3%)	

ESHAP: etoposide, methylprednisolone, high-dose cytarabine, cisplatin; R: rituximab; DICE: dexamethasone, ifosfamide, carboplatin, etoposide.

Regarding disease status, both conventional and fractionated DICE as well as ESHAP regimens had possibility to response across subgroup of patients with primary refractory disease, early and late relapse disease (Table 3).

**Table 3.** Response rate of salvage chemotherapy according to relapse or refractory status.

	ORR	*p* Value	CR	*p* Value
ESHAP				
Primary refractory (*N* = 5)	3 (60.0%)	.861	0 (0%)	.969
Early relapse (*N* = 8)	3 (37.5%)		1 (12.5%)	
Late relapse (*N* = 8)	4 (50.0%)		1 (12.5%)	
DICE				
Primary refractory (*N* = 15)	7 (46.7%)	.277	2 (13.3%)	.328
Early relapse (*N* = 17)	7 (41.2%)		4 (23.5%)	
Late relapse (*N* = 17)	12 (70.6%)		3 (17.7%)	
Conventional DICE (*N* = 12)				
Primary refractory (*N* = 4)	4 (100.0%)	.250	0 (0%)	.309
Early relapse (*N* = 3)	2 (66.7%)		1 (33.3%)	
Late relapse (*N* = 5)	5 (100.0%)		1 (20.0%)	
Fractionated DICE (*N* = 37)				
Primary refractory (*N* = 11)	3 (27.3%)	.339	2 (18.2%)	.498
Early relapse (*N* = 14)	5 (35.7)		3 (21.4%)	
Late relapse (*N* = 12)	7 (58.3%)		2 (16.7%)	

ESHAP: etoposide, methylprednisolone, high-dose cytarabine, cisplatin; DICE: dexamethasone, ifosfamide, carboplatin, etoposide; ORR: overall response rate; CR: complete response rate.

### Peripheral blood stem cell collection

**Figure 4.**
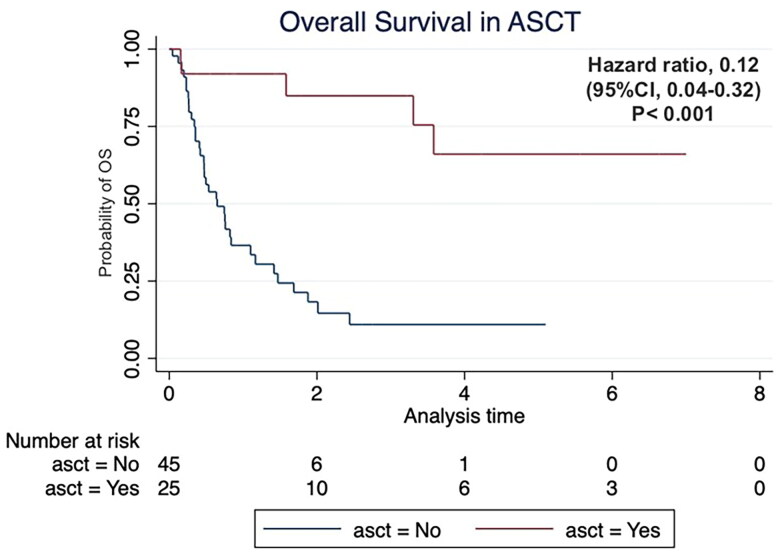
Kaplan–Meier’s curve of overall survival according to autologous stem cell transplantation (ASCT) status.

Twenty-eight patients underwent stem cell collection, including eight patients in the ESHAP group and 20 patients in the DICE group. Twenty-five patients underwent successful ASCT including six patients in the ESHAP group and 19 patients in the DICE group. The rest three patients did not receive ASCT due to lost follow-up. The survival data at the last follow-up time are shown in Table 4. The patients who received ASCT had significantly higher OS (hazard ratio (HR) 0.12; 95% CI 0.04–0.32, *p* < .001) as shown in Figure 4. The median CD34+ cell count was 11.4 × 10^6^ cells/kg (range 2.87–36.5 × 10^6^) in the ESHAP group and 4.7 × 10^6^ cells/kg (range 2.25–16.7 × 10^6^) in the DICE group (*p* = .25).

**Table 4.** Survival rate at the last follow-up time according to autologous stem cell transplantation status and salvage chemotherapy.

	Total ESHAP (*N* = 21)		Total DICE (*N* = 49)	
	ESHAP alone (*N* = 17)	R-ESHAP (*N* = 4)	DICE alone (*N* = 37)	R-DICE (*N* = 12)
Undergoing ASCT				
Yes	6/17 (35.3%)	0/4 (0.0%)	12/37 (32.4%)	7/12 (58.3%)
No	11/17 (64.7%)	4/4 (100.0%)	25/37 (67.6%)	5/12 (41.7%)
Survival rate (PFS)				
With ASCT	5/6 (83.3%)	**–**	9/12 (75.0%)	5/7 (71.4%)
Without ASCT	1/11 (9.1%)	0/4 (0.0%)	2/25 (8.0%)	2/5 (40.0%)
Survival rate (OS)				
With ASCT	5/6 (83.3%)	**–**	10/12 (83.3%)	5/7 (71.4%)
Without ASCT	3/11(27.3%)	1/4(25.0%)	4/25 (16.0%)	2/5 (40.0%)
Median time (months)				
PFS	7.7	1.8	4.7	42.9
OS	7.7	2.7	17.0	42.9

ESHAP: etoposide, methylprednisolone, high-dose cytarabine, cisplatin; R: rituximab; DICE: dexamethasone, ifosfamide, carboplatin, etoposide; ASCT: autologous stem cell transplantation; PFS: progression-free survival; OS: overall survival.

Four out of the 25 patients who received ASCT received plerixafor including one patient from the ESHAP group (12.5%) and three patients from the DICE group (15%) due to total CD34 cell counts of less than 2 × 10^6^ cells/kg in the initial stem cell collection (mobilization failure).

### Safety and adverse events of treatment

Table 5 shows the most frequent hematologic and non-hematologic AEs from both groups. Grade ≥3 anaemia was found in 14.3% of the ESHAP group whereas grade ≥3 neutropenia and thrombocytopenia were detected in 4.1% of the DICE group. Infection, febrile neutropenia and renal impairment were frequent non-hematologic AEs. Patients in the ESHAP group had more all-grade renal impairment than the DICE group (23.8% vs. 6.1%, *p* = .047). The rate of grade ≥3 infections (9.5% vs. 14.3%) and febrile neutropenia (28.6% vs. 20.4%) was comparable between ESHAP and DICE.

**Table 5.** Adverse events.

Adverse events	ESHAP (*N* = 21)		ICE (*N* = 49)	
	All grade	Grade ≥3	All grade	Grade ≥3
Hematologic adverse events				
Anaemia	3 (14.3%)	3 (14.3%)	2 (4.1%)	2 (4.1%)
Neutropenia	1 (4.8%)	–	4 (8.2%)	–
Thrombocytopenia	1 (4.8%)	–	3 (6.1%)	2 (4.1%)
Non-hematologic adverse events				
Infection	3 (14.3%)	2 (9.5%)	12 (24.5%)	7 (14.3%)
Febrile neutropenia	6 (28.6%)	6 (28.6%)	10 (20.4%)	10 (20.4%)
Renal impairment	5 (23.8%)	3 (15.8%)	3 (6.1%)	1 (2.1%)

### Subgroup analysis

**Figure 5.**
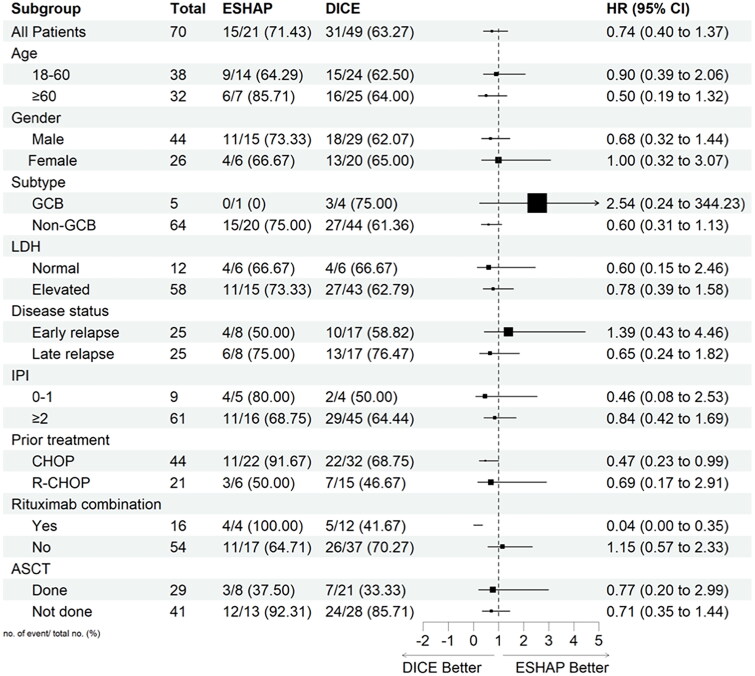
Subgroup analysis for progression-free survival.

**Figure 6.**
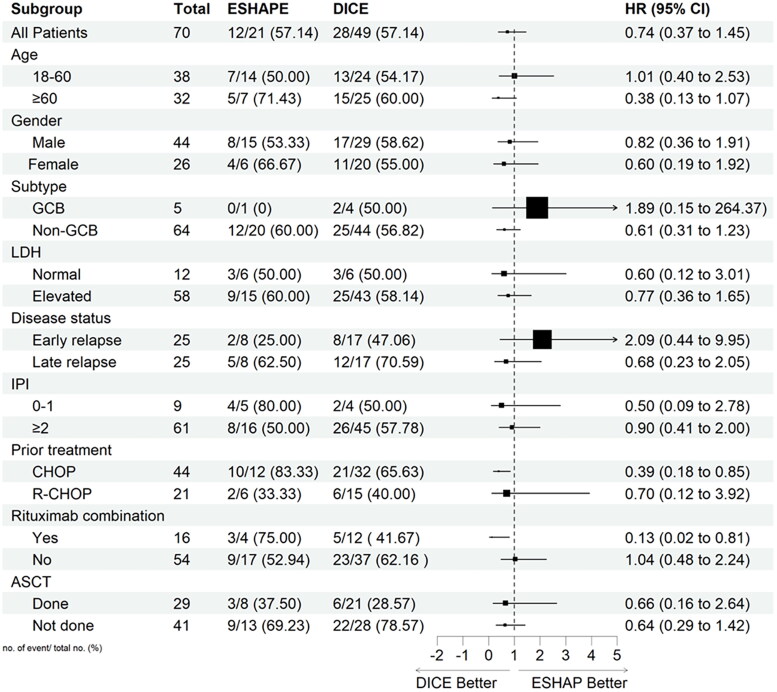
Subgroup analysis for overall survival.

PFS and OS were also assessed in exploratory subgroups defined according to patients and disease characteristics, previous therapies, and ASCT status (Figures 5 and 6, respectively). The analysis showed that patients who had received previous CHOP treatment and rituximab combination had a significant benefit in PFS and OS with DICE compared to ESHAP. The HR for previous CHOP treatment was 0.47 (95% CI, 0.23–0.99) and 0.39 (95% CI, 0.18–0.85) for PFS and OS, respectively. Similarly, HR for the rituximab combination was 0.04 (95% CI, 0.00–0.35) and 0.13 (95% CI, 0.02–0.81) for PFS and OS, respectively.

## Discussion

Relapsed or refractory DLBCL has a poor prognosis, especially in refractory patients. Regarding the large international cohort, the ORR of primary refractory disease and disease that relapsed within 12 months was only 26% with a median OS of 6.3 months [15]. On the contrary, the median OS of late relapsed DLBCL patients after 24 months was 29.9 months [16]. Overall, approximately 50% of patients with relapsed or refractory DLBCL have a response to salvage therapy and have a cure rate of about 25–35% after ASCT [1].

There are various platinum-based regimens for salvage chemotherapy for DLBCL. However, few randomized controlled trials compared the efficacy of each regimen. DHAP regimen was previously compared with ICE and GDP in the CORAL study (in combination with rituximab) [9] and NCIC-CTG LY.12 study [8], respectively. Both trials showed that DHAP had comparable efficacy in terms of ORR, PFS and OS but had more toxicities.

The ESHAP regimen has a lower dose of cytarabine compared with the DHAP regimen and was widely used in Thailand [13,17]. The ICE regimen was an emerging alternative therapy because it can be administered on an outpatient basis [10]. As a result, this study aimed to compare the efficacy and safety of these two regimens with dexamethasone added in ICE according to institutional guidelines (DICE). Accordingly, the ORR for ESHAP and DICE groups was comparable (47.6% vs. 53.1%, respectively). The ORR for the ESHAP regimen was comparable to the previous study, which reported an ORR of 46% [7]. However, ORR for the DICE regimen in this study was lower than the result from the previous phase II [10] and phase III study [9] of ICE, which showed an ORR of 85% and 63.5%, respectively. It could be explained by the differences in demographic data (such as age or eligibility for ASCT), rituximab combination, as well as the proportion of refractory and relapsed patients. A previous study showed that rituximab in combination with ICE (R-ICE) could increase the CR rate compared with ICE alone (55% vs. 28%) [4]. Since only a small proportion (24.5%) of patients in this study were able to receive rituximab due to limited access in Thailand, it might result in a lower ORR of DICE in this study. In addition, the benefit of dexamethasone combination with ICE as a DICE regimen in terms of ORR was not demonstrated in this study. Regarding fractionated DICE, the ORR of this method of administration was lower than conventional DICE (40.5% vs. 91.7%). Caution should be used for interpretation because the proportion of patients who received a rituximab combination in conventional DICE was higher than in fractionated DICE (50% vs. 16.2%).

Hematologic and non-hematologic AE rates showed no significant difference in each regimen except patients in the ESHAP group had more renal impairment than the ICE group. This finding supported the database of the Japanese Adverse Drug Event Report that showed a higher risk of renal impairment of cisplatin compared with carboplatin and another platinum-based compound (reporting odds ratio 2.7, 95% CI 2.5–3.0) [18].

Regarding stem cell mobilization, both ESHAP and ICE had similar rates of mobilization failure that required plerixafor (12.5% and 15%, respectively). This finding was consistent with other studies that reported a rate of mobilization failure up to 28% and 10–14% in ESHAP [19] and ICE [4,9], respectively. Although only 36% of patients in this study could proceed to ASCT, this group of patients had significantly better OS. The low rate of ASCT in this study led to lower PFS and OS of relapsed or refractory DLBCL in both ESHAP and DICE groups. The two-year PFS and OS in the ESHAP group in this study was only 14.3% while a GEL/TAMO study showed a five-year PFS and OS of 38% and 50% when used R-ESHAP as salvage chemotherapy and ASCT was done in about 60% of patients [20]. The two-year PFS and OS of the DICE group seemed to be higher than ESHAP in this study (26.5%) but this rate was still lower than the CORAL study, which showed a three-year PFS of 31% and three-year OS of 47% in R-ICE arm and 52% underwent ASCT. The increased accessibility for ASCT in Thailand will improve the outcomes of relapsed or refractory DLBCL in the future.

The research had limitations due to its retrospective design with a small population. It might lead to incomplete data, especially about AEs occurrences during each chemotherapy cycle. The significant difference of number of patients in two treatment groups might be concerned. The addition of dexamethasone in the ICE regimen in this study could be considered when comparing the data with other studies using ICE as well as a lower number of patients who received rituximab and underwent ASCT. Nevertheless, the results of this study represented the real-world outcomes of patients with relapsed or refractory DLBCL. In addition, data regarding efficacy of salvage chemotherapy are still important even in the era of novel treatments such as chimeric antigen receptor T-cell therapy, which have a major role in third-line therapy [1], and emerging role in primary refractory or early relapsed patients [21].

## Conclusions

The study found that ESHAP and DICE chemotherapy regimens did not show a statistically significant difference in the two-year PFS, two-year OS and ORR. However, the DICE regimen appeared to have a more favourable toxicity profile with fewer renal AEs than ESHAP and can be administered on an outpatient basis.

## Data Availability

The data that support the findings of this study are available from the corresponding author, [ER], upon reasonable request.
